# Lesbian motherhood and mitochondrial replacement techniques: reproductive freedom and genetic kinship

**DOI:** 10.1136/medethics-2017-104450

**Published:** 2018-02-28

**Authors:** Giulia Cavaliere, César Palacios-González

**Affiliations:** 1 Department of Global Health and Social Medicine, King’s College London, London, UK; 2 Centre of Medical Law and Ethics, The Dickson Poon School of Law, King’s College London, London, UK

**Keywords:** genetic engineering, history of health ethics/bioethics, gene therapy/transfer, children

## Abstract

In this paper, we argue that lesbian couples who wish to have children who are genetically related to both of them should be allowed access to mitochondrial replacement techniques (MRTs). First, we provide a brief explanation of mitochondrial diseases and MRTs. We then present the reasons why MRTs are not, by nature, therapeutic. The upshot of the view that MRTs are non-therapeutic techniques is that their therapeutic potential cannot be invoked for restricting their use only to those cases where a mitochondrial DNA disease could be ‘cured’. We then argue that a positive case for MRTs is justified by an appeal to reproductive freedom, and that the criteria to access these techniques should hence be extended to include lesbian couples who wish to share genetic parenthood. Finally, we consider a potential objection to our argument: that the desire to have genetically related kin is not a morally sufficient reason to allow lesbian couples to access MRTs.

## Introduction

One of the main purposes of bioethics is to demarcate morally acceptable applications of biomedical technologies. For example, in the past decade, there has been much debate in bioethics on whether there is a morally significant difference between therapeutic and enhancing genetic modifying interventions. ‘Bioconservatives’ such as Michael Sandel and Jürgen Habermas maintain that biotechnological practices aimed at curing disease are morally acceptable, whereas those aimed at increasing certain traits such as height and strength are morally suspicious.^1 2^ Other moral boundaries investigated by bioethicists concern morally appropriate versus inappropriate uses of reproductive screening technologies—such as preimplantation genetic diagnosis (PGD)—and of reprogenetics technologies—of which mitochondrial replacement techniques (MRTs)^i^ are an example. The latter techniques help women wishing to become mothers who carry mitochondrial DNA (mtDNA) abnormalities in their eggs to have genetically related offspring free from mtDNA diseases.^3^

MRTs have been at the forefront of bioethical debate since the UK began to discuss their legalisation in the 2000s. In February 2015, regulations were passed on two MRTs: maternal spindle transfer (MST) and pronuclear transfer (PNT). These regulations came into force in October 2015, making the UK the first country in the world to explicitly legalise MRTs under a licensed scheme.^4^^ii^

Although these technologies are legal in the UK, at the present time, only people at risk of transmitting a severe mtDNA disease can access them. The Human Fertilisation and Embryology (Mitochondrial Donation) Regulations 2015 state that the permitted circumstances for using these techniques are when:

1. There is a particular risk that any egg extracted from the ovaries of a woman named in the determination—or embryo which is created by the fertilisation of an egg extracted from the ovaries of a woman named in the determination—may have mitochondrial abnormalities caused by mtDNA.
2. There is a significant risk that a person with those abnormalities will have or develop serious mitochondrial disease.^4^

Part of the rationale for these regulations is to allow couples at risk of transmitting mtDNA diseases to have children who are free from them.^5^ In addition, MRTs may aid lesbian couples, and couples where both members have functional ovaries (ie, couples or relationships where one member may be intersex or transgender) to have genetically related children.^iii^ It has also been theorised that they can be used to increase the chances of avoiding embryonic arrest and thus allow couples whose infertility is not related to mtDNA mutations to have genetically related children too, but *this possibility awaits empirical demonstration*.^iv^ These two potential applications of MRTs are not at present legal in UK. However, it must be said that it seems that the MRTs regulations were not written down with the explicit intention of singling out these possibilities as illegal but rather in the attempt to make MRTs legal in order to avoid mtDNA diseases.^5^

Legal scholars, bioethicists and stakeholders participating in the debate on MRTs have tried to establish a morally significant boundary between acceptable and unacceptable applications of these techniques. For example, the mitochondrial disease community (patients, researchers and clinicians) have strongly advocated for a therapeutic (ie, acceptable) and a non-therapeutic (ie, unacceptable) demarcation of MRTs. By doing so they aim, in part, to avoid challenges from slippery-slope type arguments that allowing MRTs would then lead to ‘designer babies’.

In this paper, we argue that lesbian couples who want to have children who are genetically related to both of them should be allowed access to MRTs. The paper is structured as follows. First, we provide a brief explanation of mitochondrial diseases and MRTs. Second, we show that MRTs are not therapeutic in nature and thus this feature of the techniques cannot be invoked for restricting their use only to those cases where an mtDNA disease could be ‘cured’. We then argue that a positive case for MRTs is justified by an appeal to reproductive freedom and that access to these techniques should hence be extended to lesbian couples. Finally, we consider a potential objection to our argument: namely that the desire to have genetically related kin is not a morally sufficient reason to allow lesbian couples to access MRTs.

## Mitochondrial diseases and MRTs

Mitochondria have been described as the ‘powerhouses’ of our cells. They are small structures whose main known purpose is to produce the necessary energy for cellular, organ and bodily function.^6^ They are inherited via the maternal line and have their own DNA (mtDNA), which resides outside the cell’ s nucleus. Mitochondrial diseases are a cluster of neuromuscular diseases in which symptoms vary in severity and expression and can develop immediately after birth or later in life.^7 8^ Mutations both in the nuclear DNA and the mtDNA can cause mitochondrial diseases. Deleterious mutations in the mtDNA, in each cell, can happen across all mitochondria (this is known as homoplasmy) or they can occur only in certain mitochondria (known as heteroplasmy). In this paper, we will only discuss mitochondrial diseases produced by problems in the mtDNA, referred to as mtDNA diseases.

To avoid the transmission of an mtDNA disease, two MRTs have been developed: PNT and MST. PNT requires the creation of two zygotes, through assisted reproductive techniques (ARTs), one with the gametes of the intending parents (or intending mother and a sperm donor) and the other one with a donated egg and the intending father’s (or donor’s) sperm. In this scenario, the first zygote has faulty mitochondria and the second has healthy mitochondria. On the first day after fertilisation, the maternal and paternal pronuclei are removed from both zygotes. The enucleated cell produced with the intending mother’s egg and the pronuclei which were housed in the cell produced with the donor’s egg are discarded. Afterwards, the intending parents’ (or intending mother’s and donor’s) pronuclei are ferried into the enucleated cell produced with the donor’s egg. The reconstructed zygote, which possesses healthy mitochondria, can be subsequently transferred to the intending mother or a surrogate.^9^

In MST, eggs are obtained through ARTs from an intending mother and a healthy donor. The nuclear material from the intending mother’s egg and the donor’s egg is extracted. The donor’s nuclear material and the intending mother’s enucleated egg are discarded, and the intending mother’s nuclear material is ferried into the now enucleated donor’s egg.^v^ Subsequently, the reconstructed egg is fertilised in vitro and then transferred to the intending mother or a surrogate.^6^ One of the aims of both techniques is for the donor’s healthy mitochondria to help in the development of a healthy child and to be passed down via the maternal line to subsequent generations.

At present, approximately 30 mtDNA haplogroups in humans have been described.^10^ The fact that there are so many groups is important for our discussion, as there is an ongoing debate regarding mito-nuclear interactions after MRTs. Some, for example Edward Morrow, argue that if the mitochondrial haplogroup of the egg donor is not matched to that of the intending mother this could give rise to mito-nuclear incompatibility, translating into adverse health effects for the future offspring.^11^ The last report commissioned by the Human Fertilisation and Embryology Authority (HFEA) concerning MRTs being ready for clinical practice asserted that:

The panel continues to recommend that consideration is given to mtDNA haplogroup matching as a precautionary step in the process of selecting donors (…) At present, the panel believes any risks associated with a mtDNA-nuclear DNA mismatch remain theoretical; the recent studies examining embryonic cells and stem cells generated from MST-derived and PNT-derived human embryos reported no evidence of any complications or compromise of mitochondrial function arising from unmatched mtDNA haplogroups.^12^

Prior to the advent of MRTs, women at risk of transmitting an mtDNA disease who knew about their condition had the following options: first, refraining from having children; second, turning to adoption, embryo adoption or gamete donation; third, seeking to have genetically related children after undergoing oocyte sampling to assess the risk of recurrence (an option normally available to couples who have already had an affected child) or chorionic villus sampling or amniocentesis (and then deciding for or against termination) or by using PGD. It must be noted that while adoption, embryo adoption and gamete donation guarantee that future children will not be affected by an mtDNA disease, PGD and the other techniques do not always guarantee similar results.^13^ For example, PGD is not effective when the mutations are novel or uncommon, and thus there are not enough reference clinical data available to guide the couple’s decision.^13^

Different reproductive options are currently available for lesbian couples.^14^ Some of them, such as adoption, embryo adoption and gamete donation, entail either refraining from having genetically related children (adoption and embryo adoption) or having children that are genetically related to only one of the couple (third-party reproduction). Recently, another possibility, ROPA (reception of oocytes from partner), has gained some visibility.^15 16^ ROPA allows lesbian couples to have a child who is genetically related to one mother (ie, the mother who provides the oocytes which are subsequently fertilised with donor sperm) and who is gestationally related to the other mother. These options *do not allow* lesbian couples to have children who are genetically related to *both* of them.^vi^ MRTs, on other hand, would allow both women in a lesbian couple to share a genetic link with their offspring. Specifically, one of them would contribute with nuclear DNA and the other with mtDNA. Finally, it is important to mention that worldwide reproductive options for lesbian couples (and homosexual couples more generally) are often directly or indirectly limited by laws and regulations which restrict access to adoption and third-party reproduction.

## Are MRTs therapeutic in nature?

Debates on the ethics of reprogenetics technologies stir controversies as they touch on values and beliefs on the meaning of parenthood, the moral status of early human life and our obligations to future generations. In particular, debates on the ethics of introducing a new reproductive technology are characterised by reflections on the welfare of children born due to that technology. They are centred on the necessity of balancing uncertainties regarding the possible benefits and risks of such new technology and on the extent to which the reproductive freedom of prospective parents ought to be respected.^17^ Even though competing moral views generate diverging assessments of the importance that should be granted to the values and beliefs at stake, concerns related to the welfare of future children are often considered more important than the reproductive freedom of prospective parents. This is so as preventing a child (although a future one) from suffering harm is considered a morally appropriate reason to restrict prospective parents’ freedom.

Unsurprisingly, the debate on MRTs is no exception and welfare of the child considerations has been at the forefront of the ethical debate concerning these techniques. Interestingly, the welfare of children born due to MRTs has been employed *both as a critique of these techniques and as an argument in favour of them*. For instance, those who use the welfare of the future child to *oppose* MRTs maintain that these techniques are too risky for the health of future children, that their safety has not been thoroughly assessed, and that there may be unforeseen negative effects for the children conceived due to MRTs and for these children’s children.^18–23^ For example, Françoise Baylis asserts that:

Mitochondrial replacement technology is experimental and there is very limited information about safety and efficacy. As with any germline intervention, there are significant and legitimate concerns about the health and well-being of future children and the potential short-term and long-term harms to them and their progeny.^18^

According to this view, a concern for the welfare of future children (and those children’s children) warrants banning or heavily restricting MRTs until all the above-mentioned worries have been dispelled. Interestingly, many of those in favour of the techniques have also appealed to welfare of the child considerations and maintain that it is such concerns which should motivate their approval, although their take on the present safety of the techniques is radically different.^24–27^ According to such commentators, the severity of certain mtDNA conditions and their disabling and life-limiting character are sufficient reasons to allow for the clinical use of MRTs. For example, Arthur Caplan argues that an MRT procedure ‘is not without its risks, but it’s treating a disease’.^28^ And that ‘[t]hese little embryos, these are people born with a disease, they can’t make power. You’re giving them a new battery. That’s a therapy’.^28^ Framed in this way, it is clear that MRTs can be regarded as a therapy for mtDNA diseases.

The argument in favour of MRTs based on their ‘therapeutic’ nature is a powerful one: who would dare object to the approval of safe techniques that spare children from suffering? This argument runs something like this: we are morally required to prevent the suffering and premature death of innocent individuals. MRTs can prevent the suffering and premature death of existing innocent individuals. Hence, we are morally required to carry out MRTs.

The framing of MRTs in terms of a therapy for mtDNA diseases for existing individuals (in contrast with future ones) allows supporters of these techniques to build a moral case in favour of their approval and, at the same time, to raise a supposedly justified moral boundary. The moral line is drawn between uses that are therapeutic, and hence good, and uses that are ‘beyond therapy’, and hence morally suspicious. In order to make our case that lesbian couples should have access to MRTs to have genetically related children, we first challenge their alleged therapeutic nature. Doing so allows us to show that the therapeutic/non-therapeutic moral boundary does not exist and thus that criteria of access to MRTs must be grounded on other considerations.

Thus far, Wrigley *et al*^27^ have carried out the most thorough defence of the therapeutic nature of MRTs (or at least of one of the two techniques). The authors maintain that ‘PNT […] is a form of therapy based on embryo modification while MST is, instead, an instance of selective reproduction’.^27^ They draw this conclusion from the observation that the process of PNT (which entails enucleation, transfer and reconstitution) does not affect the numerical identity of the embryo as it already exists. PNT pre-emptively cures an already existing being. Conversely, at the point of the process of MST (which also entails enucleation, transfer and reconstitution), it is unknown (in almost all cases) which *sperm cell* will fertilise the reconstituted oocyte, and thus the identity of the future individual has not been determined (supposing that our numerical identity is determined by specific gametes which fuse). On this basis, Wrigley *et al* conclude that MST cannot cure anyone while PNT does. The upshot of their argument is that there ‘is a strong prima facie harm-avoidance rationale for *offering* PNT to prospective parents and for those parents to accept it; one that is not present in the case of MST [emphasis added]’.^27^

Wrigley *et al*’s stance has been criticised for a number of reasons.^29 30^ One point of contention is that there is no harm-avoidance rationale *for offering PNT* to prospective parents, as at the point of offering it there is no one who could be subject to PNT and thus no one who could be cured. When *the clinical decision to employ* PNT is made, it affects which sperm and egg will fuse, which means that: ‘the gametes that will fuse in order for the process of PNT to happen *would most certainly not have fused* in the first place if PNT had not been chosen as the course of action’.^29^ This is the case because after the decision to carry out PNT has been made, the woman will have to be subject to hormonal stimulation and to the egg extraction process. This means that the egg that would have been fertilised the month that she/the couple decided to undergo PNT is not the same egg as that which will be fertilised prior to undergoing the PNT procedure. And even in the rare case of having only one single cryopreserved egg, the sperm cell that will fertilise the egg will depend on when the sperm sample is provided or which sperm from an already collected sample is actively chosen or which sperm happens to fertilise the egg in vitro from an already collected sample. All this shows that *the clinical decision to employ* PNT affects the timing of conception and thus who will exist.

Additionally, Matthew Liao has argued from an Organism View account that *the process of MST and PNT* is numerically identity-affecting.^31^ According to Liao, the enucleation, transfer and reconstitution actions are of such nature that both eggs, or both embryos, cease to exist and a *third* egg, or embryo, is created. In order to understand Liao’s argument, we must bear in mind that an egg, or embryo, is an organism. An organism, *as a kind of thing*: (1) begins to exist when the capacity to regulate and coordinate the various life processes (respiration, absorption, metabolism and so on) is there; (2) it persists as long as there is a continuing ability to regulate and coordinate the various life processes and (3) it ceases to exist when the capacity to regulate and coordinate the various life processes is permanently gone.^31^ The two main reasons why the enucleation process permanently disrupts the organismic continuity processes of the eggs, or zygotes, are: first, that the cytoplasm of an egg, or zygote, contains crucial components for regulating and coordinating the various life processes; second, that there are life processes in the cytoplasm of an egg, or zygote, that the nucleus does not control (fully, at least).^31^ What this means is that an egg’s capacity to regulate its metabolism, for example, is destroyed when we enucleate it, and thus *a new capacity* comes into being when we transfer the intending mother’s maternal spindle into the donor’s enucleated egg. This metaphysical stance is relevant when morally assessing MRTs, as it follows from it that ‘in essence’ neither technique is therapeutic. They are not therapeutic because they *do not cure anyone;* they just bring into existence a new organism.

Furthermore, by maintaining that numerical identity follows the nuclear DNA, Wrigley *et al* appear to endorse the view that cells are essentially their nuclear genes (or a collection of them). But if *genes* are what establish numerical identity, then why is the mtDNA not part of what constitutes the numerical identity of a cell, as it also contains genes? Why consider only the nuclear genome and not that plus the mitochondrial one? And equally, why is it the case that *all* the chromosomes establish numerical identity and not only a subset of them? Wrigley *et al*’s view does not offer a compelling case of the notion that cells are essentially their nuclear genomes.

According to the previous arguments neither MST nor PNT are therapeutic and hence a moral case for them and, more importantly, for restricting their use cannot be based on how the welfare of a *particular* child will be improved. These considerations have two implications: on the one hand, it is necessary to abandon the rhetoric of cure and therapy and on the other that additional reasons should be presented to ground the moral case in favour of MRTs. Let us now consider another argument that could justify the moral acceptability of MRTs: reproductive freedom.

## Reproductive freedom and MRTs

Those who have advocated the legalisation of MRTs in UK have frequently appealed to the importance of allowing couples at risk of transmitting an mtDNA disease, the freedom to choose to procreate according to their preferred life plan: what is commonly referred to as reproductive freedom or procreative liberty.^vii^^32–35^ They argue that couples should be free to choose whether to have genetically related healthy children and that third parties—be them the state, religious institutions or fellow citizens—should not interfere with their choices. For example, Andrew Miller, the chair of UK’s Commons Science and Technology Committee from 2010 to 2015, argued against the lobbying efforts by religious groups to reject MRTs: ‘It is utterly outrageous in a free society for the churches to tell parents who are in this painfully difficult position that they cannot undergo procedures like this’.^36^ Why was Miller angered by the churches’ interference in procreative decisions? In this section, we first try to make sense of Miller’s (and other defenders of reproductive freedom) outrage, and we then show that if MRTs fall within the remit of the reproductive freedom of heterosexual couples where women are at risk of transmitting an mtDNA disease, then they also fall within the remit of the reproductive freedom of lesbian couples.

In contemporary Western democratic societies, freedom of choice is defended from third parties’ interference on political and moral grounds. This has its roots in the work of John Stuart Mill and other liberal philosophers. Mill believed that the only appropriate moral ground for interference in one’s actions is if one’s free agency may cause *harm to others*.^32 37^ In *On Liberty*, he asserts that there should only be ‘one very simple principle, as entitled to govern absolutely the dealings of society with the individual in the way of compulsion and control’. The principle states that:

the only purpose for which power can be rightfully exercised over any member of a civilised community, against his will, is to prevent harm to others. His own good, either physical or moral, is not a sufficient warrant.^37^

The former is commonly known as Mill’s ‘Harm Principle’, a principle that sits at the core of our liberal democratic societies, where, ‘the presumption in favour of the freedom of citizens to make their own choices without interference places the burden of proof on attempts to limit freedom’.^38^ Isaiah Berlin labelled this Millian understanding of freedom as *negative freedom* or *freedom from*.^39^ Elements of this negative understanding of freedom survive in defences of the moral right of people to make ‘autonomous choices in matters of procreation’^40^ or, as John Robertson puts it: ‘the freedom to reproduce or not to reproduce in the genetic sense’.^33^ John Harris, John Robertson, Dan Brock and other contemporary advocates of reproductive freedom strongly emphasise the importance of defending the freedom of people to make significant choices in matters of procreation without third parties’ interference. They also maintain that this procreative freedom ought to be limited only if it becomes incompatible with a like liberty for all or if it may cause significant harm to others. Harris’ and Robertson’s theorising of reproductive freedom only in negative terms has been criticised most notably by Catherine Mills, who argues that reproductive freedom also contains positive elements and who understands it as a ‘practice of self-making’, one that allow prospective parents to ‘give shape’ to their lives.^38^ In this sense, reproductive freedom incorporates the negative elements of the Millian liberal tradition and some of the positive elements that Berlin also identified, those that allow for self-determination and that make our actions the product of our own agency.^39^

But why does reproductive freedom matter? Why is it a constant reference and point of contention in debates on assisted reproduction? Different authors have provided (slightly) different accounts of why reproductive freedom ought to be treated as a fundamental moral good, but at the core of all these accounts are two moral bases for its defence: the centrality of reproduction for the development of personal life plans (the autonomy argument for reproductive freedom) and for the well-being of individuals (the welfarist argument for reproductive freedom). The autonomy argument grounding reproductive freedom refers to the morally relevant interest of individuals shaping their own lives according to the values or interests which are relevant to them.^41 42^ Reproductive freedom is thus important not in itself but due to ‘the values or interests or standing that this particular constraint defeats’.^41^ Applied to the MRTs debate, the autonomy argument provides a sound moral defence of the right of couples at risk of transmitting an mtDNA disease to their children to reproduce as they want and to have healthy children that are genetically related to them. The welfarist argument, on the other hand, focuses on the relevance of reproductive decisions for individuals’ well-being and understands reproduction as a ‘core human activity’^33^ or ‘fundamental right’.^43^ Failing to respect reproductive freedom and placing constraints on its exercise may negatively impact individuals’ well-being and their ability to lead a good life.^35^ It is for these reasons that reproductive freedom should not be interfered with for *trivial* reasons and that placing limits on reproductive freedom is morally acceptable only for significant reasons, such as the occurrence of significant harm to others.^viii^

When we take into consideration our previous discussion on the ‘therapeutic’ nature of MRTs, we realise that Mill’s ‘harm principle’ does not relate to a consideration of the created child. What we are maintaining here is that under a personal account of morality and a counterfactual account of harm—‘if your act harms someone, then it makes that person worse off than they would have been had you not done the act’^44^—neither PNT nor MST leave created children worse off than they would otherwise have been. Such children are not made worse off by MRTs because the only other available ‘option’ for them is not to exist.^ix^

Our premise that MRTs do not inflict harm to future children leads to the conclusion that these technologies fall, under a Millian understanding of freedom, within the proper remit of the reproductive freedom of women with mtDNA diseases. Given the moral importance of reproductive freedom for people’s capacity to be autonomous and for their well-being, we can further argue that *the current UK* legislation on MRTs benefits women at risk of transmitting an mtDNA disease (and their partners). It benefits them as these techniques represent an additional reproductive option, one that allows them to have healthy genetically related children (if they wish to do so).^x^ Then again, (explicitly) *legislating against* MRTs would violate these women’s reproductive freedom by restricting their *significant* range of reproductive options and the possibility of enjoying genetic parenthood. The upshot of considering that the moral case in favour of these technologies is that they add a significant reproductive option to prospective parents is that the ethical focus shifts from mainly taking into account questions of the safety and welfare of future children to considering how these technologies have the potential to *benefit* prospective mothers and couples.

At this point, we have reached the crux of the issue: namely, the moral reasons for making MRTs available to women at risk of transmitting an mtDNA disease, ceteris paribus, also ground their access to lesbian couples as: (1) people have a great interest in reproduction because of how it shapes their lives according to the values and interests which are relevant to them, and it is also a very deep personal and private project which has a significant impact on individuals’ well-being and (2) the fact that MRTs cannot be said to harm any child created through their use. Finally, the fact that lesbian couples need a sperm donor, in addition to their own eggs, does not detract from our stance. It does not do so as sperm donation for family-making purposes is morally acceptable.^45^

At this point, it would be possible to counter that mitochondria *only* produce energy and *only* represent 1% of the total amount of genetic material, and thus that lesbian couples opting for them would just be embarking on a very expensive vanity project. Explaining in detail why these claims, which John Appleby^46^ has named the ‘qualitative claim’ and the ‘quantitative claim’, are problematic for arguing that MRTs cannot establish parenthood would require much more space than we have available here.^47^ What we can state is that, following our previous section on how MRTs affect numerical identity, in the case of a lesbian couple both mothers would be parents under a causal account of parenthood, at least. They would be so because: ‘any [free] action that reasonably foreseeably results in the birth of a child generates responsibilities for that child’.^48^ And in this case, their free action of seeking MRTs, and the subsequent assisted reproductive steps, reasonably foreseeably results in the birth of a child.

## Reproductive freedom and treating like cases alike

The possible use of MRTs as a reproductive option by lesbian couples has already been mentioned in the bioethics literature by the Nuffield Council on Bioethics, Françoise Baylis, Palacios *et al*, Rebecca Dimond, Ishii and Segers *et al*.^18 49–54^ Furthermore, from a legal point of view, Danielle Griffiths has explored how UK regulations on MRTs reproduces the heteronormative genetic family.^55^

However, in such literature, this possible application of MRTs is typically mentioned only in passing. A notable exception is Françoise Baylis. In her article ‘The ethics of creating children with three genetic parents’ she lists this possible use of MRTs under the heading ‘Harms to society’. She asserts:

While the initial goal of mitochondrial replacement technology is ‘therapeutic’ insofar as it aims to avoid the birth of a child with mitochondrial disease, this technology could be used without therapeutic intent. For example, it could be used to pursue non-therapeutic reproductive goals—imagine, a lesbian couple where both partners wanted a genetic link to the children they intend to parent.^18^

Why the use of MRTs by lesbian couples would be harmful to society remains unclear in her article. With some exercise of imagination, and assuming that she in fact believes so, it seems that such harm stems from the fact that this use of MRTs would not be ‘therapeutic’, understanding therapeutic in the sense that ‘it aims to avoid the birth of a child with mitochondrial disease’.^18^ Non-therapeutic uses of technologies have been frequently condemned by bioethics scholars because they may corrupt values that we cherish^2^; they may damage our relationships among members of a society of equals^1^ and they may be instances of eugenics.^56^ However, despite Baylis’ concerns, morality demands treating like cases alike: if we accept that the use of MRTs by women at risk of transmitting an mtDNA disease neither harms society because a child without a mitochondrial disease would be created nor spares any individual from suffering, then we have to accept that the use of MRTs by lesbian couples does not harm society, because a child without a mitochondrial disease would be created, nor spares any individual from suffering. It is true that both types of uses could be considered ‘eugenic’, rather than ‘therapeutic’, in the sense that they aim to bring a particular kind of individual into existence: healthy people who are genetically related to their parents. It is for the above-mentioned reasons that we find Baylis’ position wanting. All the more so, denying access to MRTs to lesbian couples is ethically unjustifiable in as much as it curtails the enjoyment of certain freedoms to a certain group without good reason, while allowing others to enjoy the very same freedoms. Those who want to prohibit the use of MRTs by lesbian couples need to present an argument for showing that them obtaining access to this technology is unethical, an argument that so far no one has successfully presented.^xi^

## Genetic relatedness and MRTs

Let us take stock of what we have argued thus far. We have presented some arguments against the view that MRTs are *therapeutic* technologies and hence concluded that concerns for the welfare of the future child cannot ground their moral acceptability nor restrict their use. We have then focused on the other reason that may morally justify offering MRTs, namely the reproductive freedom of prospective parents. We have argued, *contra* the position of those who want to restrict use of MRTs only to women at risk of transmitting mtDNA diseases, that morality demands treating like cases alike; and we maintain that a concern for equality would deem immoral a restriction on the use of MRTs based on one’s belonging to a group with certain sexual preferences. In this final section, we consider a potential objection to our argument: namely that the desire for genetic relatedness is not a morally sufficient reason to allow lesbian couples to access MRTs.

One of the criticisms against MRTs, and against other reproductive technologies, is that their sole benefit is to allow parents to have a genetic tie to their offspring, which is considered a morally dubious end.^57^ In this section, we refer to this as the genetic-relatedness objection (GRO) to MRTs. Underlying the GRO are two distinct types of concerns, one inspired by deontological concerns and the other inspired by consequentialist concerns. Deontological concerns (GRO-d) centre on the morally dubious character of those seeking genetic relatedness. This desire is suspect, critics argue, because it expresses a non-virtuous parenting attitude, one that aims at having *particular kinds* of children, which is considered by critics ‘a wish and not a need’.^2 18^ We do not explore further the GRO-d, as others have done so.^58^ Other concerns underlying the GRO are consequentialist in nature (GRO-c). GRO-c focuses on the negative consequences which allowing prospective parents to use MRTs (and other ARTs) may generate. The negative consequences identified by the critics include: concerns for the resources needed to develop new technologies and how these resources may be employed for other more pressing medical needs^18 57^; the reinforcement of ideas on the importance of genetic kinship for family-making and on the role of genetics more generally to determine our identities^57 59^; the medicalisation of a social preference^57 59^ and the reinforcement of the two-parent (heterosexual) genetically based model of the family (ie, bionormative conception of the family).^19^

At first sight, the initial type of GRO-c concerns, those hinging on the scarcity of available resources, seems to be legitimate. In practical terms, what this concern means is that, when we argue about the moral permissibility of MRTs, we have to factor in the costs of *satisfying this preference*, even if it is a strongly held one, against other medical opportunity costs, for example *satisfying the basic medical needs of others*. According to Baylis, once faced with this choice we have to reach the conclusion that research and clinical practice on MRTs are immoral. They are immoral given that they use scarce medical resources that could be better used elsewhere, because, as noted by Rulli and others, the development of MRTs requires(-ed) the use of vast resources both in terms of budget and personnel.^57^ One way to respond to this objection is to note that even if we grant Rulli’s and Baylis’ point regarding the use of scarce medical resources, from this fact it *does not inherently follow* that the use of medical scarce resources for MRTs is immoral. This is because in order to make such a claim, we need to prove that when compared against all other medical research that is being carried out the use of scarce medical resources for MRTs is unwarranted.^60^ Our concern here is *not* to examine the ethical case in favour of or against MRTs nor to provide an account of the ethical issues surrounding these techniques, but rather to stress the need to *extend the existing criteria of access to these techniques to lesbian couples*. Furthermore, concerns related to the necessary clinical research to develop MRTs do not apply in the case of lesbian couples, as these techniques are already in place; and in fact the use of MRTs by lesbian couples, and possibly by other non-mtDNA infertile couples, should be factored in when considering the overall offsetting of the costs of this research.

Last, we consider GRO-c concerns related to the reinforcement of genetic deterministic ideas about the importance of genetic relatedness for family-making and the reinforcement of the bionormative family. Many women and couples have a strong preference for having genetically related children.^xii^^61^ This is true for both women with mtDNA diseases and lesbian couples, and we contend that in a liberal society allowing only heterosexual couples to enjoy the satisfaction of their wish, regardless of its philosophical validity, is problematic from the point of view of equality. This is akin to only allowing certain ethnic groups to access assisted reproductive technologies, for example.

In addition, gay and lesbian couples’ reproductive choices are already limited: depending on the countries’ regulations, these couples are often ineligible for third-party reproduction and for adoption. Preventing them from using an already existing technology due to consequentialist concerns related to the reinforcement of genetic determinist ideas on the value of genetic relatedness seems to us akin to further restricting their already limited agency with respect to reproductive options.^xiii^ Hence, even though it is true that we should be attentive to the fact that MRTs could contribute to increasing the value attributed to genetic relatedness, to the detriment of other forms of family-making, it must be said that it would be morally problematic to just focus on lesbian couples and their wishes and choices thereof. In other words, we believe that it is compatible to hold the view that reproductive technologies such as MRTs might have undesirable consequences such as the ones described by the critics of these technologies, and the view that genetic relatedness seems to be an important good whose enjoyment should not be restricted on an arbitrary basis.

Regarding GRO-c concerns for the preservation of the bionormative family, it must be noted that in the case of MRTs being used by lesbian couples, this charge does not apply. The use of MRTs by lesbian couples in fact defies the current dominion of the bionormative family in that it challenges the *folk assumption* about the *correct type and amount* of shared genes that are necessary for establishing a parental genetic link—50% of the nuclear genes from the father and 50% of the nuclear genes from the mother.^xiv^^47^ Specifically, what it is asserted here is that 1% of an mtDNA genetic connection *suffices* for establishing genetic parenthood.^xv^ Even more so, regulating MRTs so as to include lesbian couples would expand the models of *state-recognised* genetic relatedness and challenge the existing order and, as seen by Griffiths, not doing so would be ‘an example of how science and regulation seek to expand models of traditional relatedness in a way that does not challenge the (bionormative) existing order’.^55^

## Conclusion

In this paper, we have challenged the view that MRTs are a therapy for mitochondrial diseases, and that these techniques can be considered harmful to children. We have argued that the rationale for offering these techniques must lie somewhere else, namely within concerns for the reproductive freedom of prospective parents. Shifting the focus of the moral debate on MRTs from concerns for the welfare of the children to other moral justifications for offering MRTs allows for the emergence of other questions that require moral consideration. In particular, it allows us to consider how an unduly restrictive approach to accessing MRTs to a particular group requires arguments that have not been presented thus far. We do not want to defend here the wish for genetic kinship as an absolute good that trumps other considerations and nor do we believe that reinforcing a family-making process that includes a genetic element is without costs. However, we remain convinced that these considerations cannot be employed solely to bar access to MRTs by lesbian couples, a group with an already limited range of reproductive options, as this would be immoral from an equality standpoint.

## Footnotes

i Even though the name ‘mitochondrial replacement techniques’ is contested, here we use it because it has secured a foothold within the academic debate; see Palacios-González’s *Mitochondrial replacement techniques: egg donation, genealogy and eugenics*.^62^ Ainsley Newson and Anthony Wrigley have recently proposed and defended the term ‘mitochondrial targeting techniques’.^63^

ii Interestingly, the first couple of babies born because of MRTs were not born in UK, but in USA (by means of maternal spindle transfer) and Ukraine (by means of pronuclear transfer).^64 65^

iii Even when in this paper we mainly refer to lesbian couples our arguments equally apply to all couples where both members have functional ovaries or cryopreserved eggs.

iv Due to space constraints, we do not investigate, or focus on, the ethical aspects of offering MRTs to heterosexual couples with non-mtDNA-related infertility problems. We also do not discuss other ethical issues related to MRTs more generally, such as risks to egg providers.

v Both for PNT and MST, if during the chromosomal transfer there is a large unintentional carryover of pathological mitochondria the mtDNA disease could manifest immediately afterwards and in subsequent generations.^66 67^

vi Even when it is true that there are epigenetic influences during pregnancy and that the gestating mother could be thought to have genetic ties to the child she bears, here we are using ‘genetic’ in a more narrow sense. We understand ‘genes’ as physical tokens of transmission that originate in the parent and that could be said to materially overlap between parent and child. We appreciate that there are many and important philosophical questions regarding the role of epigenetic influence in parenthood, but they are beyond the scope of this paper. We are indebted to one of the anonymous reviewers for pointing this out to us.

vii Here we do not distinguish between the different expressions used to refer to reproductive freedom (ie, reproductive autonomy and procreative liberty).

viii For a discussion of the limits of reproductive freedom and of limits other than significant harm to others, see Dan Brock and Allen Buchanan *et al*, chapter six in particular.^32 35^

ix Two things must be clear: first, that the only case where someone could be harmed here is if their life is a wrongful one; second, that this is a classic instance of the Non-identity Problem. Expanding on the Non-identity Problem would require more space than is available here. For a compelling case of why children are not harmed, see David Boonin’s work on this issue.^44 68^

x The idea that more choices lead to greater freedom and well-being has been challenged notably in the work of Gerald Dworkin, Nikolas Rose and Barbara Katz Rothman.^69–71^ They have argued that more options can also have the effect of bringing about more perceived and actual responsibilities. Recently, one of us has further elaborated this view in the context of genome editing and assisted reproduction.^17^ In the case of MRTs and couples at risk of transmitting an mtDNA disease, however, the range of (reproductive) options currently available includes either refraining from having offspring who are genetically related to both prospective parents or risking passing on the mtDNA disease. MRTs would allow the additional option of having healthy children who are genetically related to both prospective parents. Similarly, as shown above, the (reproductive) options currently available to lesbian couples do not allow them to enjoy genetic kinship. For these reasons, MRTs could not be said to count as a *mere quantitative* addition to the range of options currently available to prospective parents, but as a *qualitatively significant* new option. We are indebted to an anonymous reviewer for bringing this point to our attention.

xi At this point, someone might claim that the possibility of mito-nuclear incompatibility speaks against the use of MRTs by lesbian couples. We can reply to this challenge in the following way: first, there can be lesbian couples where both women belong to the same haplogroup, and thus even if such interaction-worries materialise for them, they would not be a problem. Second, at this point in time, as the HFEA report mentions, such putative problems are theoretical, and even if they were to materialise lesbian couples should still have the option of resorting to MRTs (unless the created lives were wrongful ones). This position is not a radical one, but just the same as that which postulates that couples should be able to resort to assisted reproduction even when they know that their children might be at an elevated risk of having a disability.

xii One may dispute, as previously stated, that MRTs allow couples at risk of transmitting an mtDNA disease and lesbian couples to achieve the same end, namely to have genetically related children. While for the first group of couples MRTs allow them to have children whose genetic make-up contains genetic information from both nuclei of the parents, for lesbian couples the situation is different as one party of the couple would provide the nuclear DNA and the other would provide the mitochondrial DNA. The *sociological* question of whether contributing with ‘merely’ the mtDNA would be sufficient for lesbian couples to regard the children born thanks to the aid of MRTs *as their own* is yet to be answered. However, we maintain that the legislature should not a priori rule against this, based on the idea that mtDNA contributes less to genetic kinship than nuclear DNA.

xiii From *The Human Fertilisation and Embryology (Mitochondrial Donation) Regulations 2015*, it is not at all clear if in UK MRTs can only be accessed by heterosexual couples, or if lesbian couples could access them if one of the intending genetic parents is at risk of passing on a serious form of mtDNA disease.

xiv It must be noted that in the *reproductive cloning debate*, some authors already acknowledge the possibility of becoming a genetic parent through mtDNA. Mary Mahowald, for example, asserted that ‘through use of one woman’s nuclear DNA and another woman’s enucleated egg, a lesbian couple may have a child who is biologically related to both without requiring sperm donation’ and that ‘the ovum in which that parent’s DNA is inserted represents a significant environmental influence on development, and the mitochondrial DNA adds a genetic component to the environment of the nuclear DNA’.^72^ Others who have commented on this issue are Jean Chambers, Timothy Murphy and Carson Strong.^73–76^

xv It must be noted that this claim aims at subverting the *folk western conception of genetic parenthood*, and that it is not a claim regarding the metaphysics of reproduction. For an account of why MRT-conceived children do have three genetic parents, see Monika Piotrowska’s *Is ‘Assisted Reproduction’ Reproduction?*.^77^

## References

[1] Habermas J The Future of Human Nature. Cambridge, UK: Polity Press, 2003.

[2] Sandel MJ The Case against Perfection: ethics in the age of Genetic Engineering. 1 edition Cambridge, Mass: Belknap Press, 2009.

[3] Vogel G U.K. Parliament approves controversial three-parent mitochondrial gene therapy. Science 2015 http://www.sciencemag.org/news/2015/02/uk-parliament-approves-controversial-three-parent-mitochondrial-gene-therapy (accessed 26 May 2017). 10.1126/science.aaa7793

[4] The Human Fertilisation and Embryology (Mitochondrial Donation) Regulations 2015. The Human Fertilisation and Embryology (Mitochondrial Donation) Regulations. 2015 http://www.legislation.gov.uk/uksi/2015/572/pdfs/uksi_20150572_en.pdf

[5] Scott R, Wilkinson S, Modification GG and Identity: The Mitochondrial and Nuclear Genomes. Oxf J Leg Stud 2017:1–31. Forthcoming. 10.1093/ojls/gqx012 PMC5903588 29670305

[6] Tachibana M, Sparman M, Sritanaudomchai H, et al Mitochondrial gene replacement in primate offspring and embryonic stem cells. Nature 2009;461:367–72. 10.1038/nature08368 19710649 PMC2774772

[7] Bredenoord AL, Braude P Ethics of mitochondrial gene replacement: from bench to bedside. BMJ 2010;341:c6021 10.1136/bmj.c6021 21059727

[8] Reinhardt K, Dowling DK, Morrow EH, et al evolution, and the clinic. Science 2013;341:1345–6. 24052294 10.1126/science.1237146

[9] Craven L, Tuppen HA, Greggains GD, et al Pronuclear transfer in human embryos to prevent transmission of mitochondrial DNA disease. Nature 2010;465:82–5. 10.1038/nature08958 20393463 PMC2875160

[10] van Oven M, Kayser M Updated comprehensive phylogenetic tree of global human mitochondrial DNA variation. Hum Mutat 2009;30:E386–E394. 10.1002/humu.20921 18853457

[11] Reinhardt K, Dowling DK, Morrow EH, et al Evolution, and the Clinic. Science 2013;341:1345–6. 24052294 10.1126/science.1237146

[12] Greenfield A Scientific review of the safety and efficacy of methods to avoid mitochondrial disease through assisted conception: 2016 update. UK, 2016 http://hfeaarchive.uksouth.cloudapp.azure.com/www.hfea.gov.uk/10557.html (accessed 12 Oct 2017).

[13] Poulton J, Kennedy S, Oakeshott P, et al Preventing transmission of maternally inherited mitochondrial DNA diseases. BMJ 2009;338:b94 10.1136/bmj.b94 19181733

[14] De Wert G, Dondorp W, Shenfield F, et al ESHRE Task Force on Ethics and Law 23: medically assisted reproduction in singles, lesbian and gay couples, and transsexual people. Hum Reprod 2014;29:1859–65. 10.1093/humrep/deu183 25052011

[15] Marina S, Marina D, Marina F, et al Sharing motherhood: biological lesbian co-mothers, a new IVF indication. Hum Reprod 2010;25:938–41. 10.1093/humrep/deq008 20145005

[16] Zeiler K, Malmquist A Lesbian shared biological motherhood: the ethics of IVF with reception of oocytes from partner. Med Health Care Philos 2014;17:347–55. 10.1007/s11019-013-9538-5 24395218

[17] Cavaliere G Genome editing and assisted reproduction: curing embryos, society or prospective parents? Med Health Care Philos 2017;18 10.1007/s11019-017-9793-y PMC5956052 28725950

[18] Baylis F The ethics of creating children with three genetic parents. Reprod Biomed Online 2013;26:531–4. 10.1016/j.rbmo.2013.03.006 23608245

[19] Baylis F Human nuclear genome transfer (so-called mitochondrial replacement): clearing the underbrush. Bioethics 2017;31:7–19. 10.1111/bioe.12309 27973718

[20] Morrow T Safety concerns remain over three-person IVF. The Guardian 2014 https://www.theguardian.com/science/2014/jul/22/three-person-ivf-mitochondria-dna (accessed 12 May 2017).

[21] Newman SA The British Embryo Authority and the Chamber of Eugenics. Huffington Post 2013 http://www.huffingtonpost.com/stuart-a-newman/mitochondrial-replacement-ethics_b_2837818.html (accessed 25 Jan 2016).

[22] Melo-Martin de I When the milk of human kindness becomes a luxury (and untested) good. a reply to harris’ unconditional embrace of mitochondrial replacement techniques. Camb Q Healthc Ethics 2017;26:159–65. 27934576 10.1017/S0963180116000724

[23] de deM-MI Rethinking Reprogenetics: Enhancing Ethical Analyses of Reprogenetic Technologies. 1 Edn Oxford; New York: OUP USA, 2016.

[24] Caplan A Is It Ethical to Create Babies From Three DNA Sources? Absolutely. WIRED 2015 https://www.wired.com/2015/02/ethical-create-babies-three-dna-sources-absolutely/ (accessed 12 May 2017).

[25] Harris J Germline Modification and the Burden of Human Existence. Camb Q Healthc Ethics 2016;25:6–18. 10.1017/S0963180115000237 26465828

[26] Johnson MH Tri-parenthood--a simply misleading term or an ethically misguided approach? Reprod Biomed Online 2013;26:516–9. 10.1016/j.rbmo.2013.03.014 23602720

[27] Wrigley A, Wilkinson S, Appleby JB Mitochondrial replacement: ethics and identity. Bioethics 2015;29:631–8. 10.1111/bioe.12187 26481204 PMC4620703

[28] Smith M FDA considering 3-parent embryos. CNN 2014 http://www.cnn.com/2014/02/26/health/ivf-mitochondria/index.html (accessed 13 Feb 2017).

[29] Palacios-González C Are there moral differences between maternal spindle transfer and pronuclear transfer? Med Health Care Philos 2017;20:503–11. 10.1007/s11019-017-9772-3 28429249 PMC5665963

[30] Rulli T The mitochondrial replacement ’therapy' myth. Bioethics 2017;31:368–74. 10.1111/bioe.12332 28039887

[31] Liao SM Do Mitochondrial Replacement Techniques Affect Qualitative or Numerical Identity? Bioethics 2017;31:20–6. 10.1111/bioe.12308 27973721

[32] Buchanan A, Wikler D, Daniels N From Chance to Choice: Genetics and Justice. New Ed edition. Cambridge, U.K.; New York: Cambridge University Press, 2000.

[33] Robertson JA Children of choice: freedom and the new reproductive technologies. Princeton University Press 1996.

[34] Robertson JA Procreative liberty in the era of genomics. Am J Law Med 2003;29:439–87. 15119245

[35] Brock DW Shaping future children: parental rights and societal interests. J Polit Philos 2005;13:377–98. 10.1111/j.1467-9760.2005.00229.x

[36] Mason R, Sample I, McVeigh K Church ‘irresponsible’ for trying to sway MPs against mitochondrial donation law. The Guardian 2015 https://www.theguardian.com/science/2015/feb/02/church-groups-irresponsible-pushing-mps-against-ivf-law-change (accessed 15 May 2017).

[37] Mill JS On Liberty, Utilitarianism and Other Essays. 2 Edn OUP Oxford: Oxford, 2015.

[38] Mills C Reproductive autonomy as self-making: procreative liberty and the practice of ethical subjectivity. J Med Philos 2013;38:639–56. 10.1093/jmp/jht046 24225390

[39] Berlin I Two Concepts of Liberty Oxford University Press. London: Four Essays on Liberty, 1969.

[40] Harris J Rights and reproductive choice : Harris J, Holm S, The future of human reproduction: ethics, choice and regulation. UK: Oxford University Press, 1998.

[41] Dworkin R Life’s Dominion: an argument about abortion, euthanasia, and individual freedom. Harper Collins 1993.

[42] Schaefer GO, Labude MK Genetic affinity and the right to ’three-parent IVF'. J Assist Reprod Genet 2017;34:1577–80. 10.1007/s10815-017-1046-8 28920184 PMC5714827

[43] Liao SM The Right to Be Loved. 1 Edn Oxford; New York, NY: OUP USA, 2015.

[44] Boonin D How to Solve the Non-Identity Problem. Public Aff Q 2008;22:129–59.

[45] Brandt R, Wilkinson S, Williams N The Donation and Sale of Human Eggs and Sperm Zalta EN, The Stanford Encyclopedia of Philosophy. California, USA: Metaphysics Research Lab, Stanford University, 2017 https://plato.stanford.edu/archives/sum2017/entries/gametes-donation-sale/ (accessed 23 Jun 2017).

[46] Appleby JB Should Mitochondrial Donation Be Anonymous? J Med Philos 2017 10.1093/jmp/jhx022 PMC5901087 29301011

[47] Palacios-González C Does egg donation for mitochondrial replacement techniques generate parental responsibilities? J Med Ethics 2017:medethics-2017-104400 10.1136/medethics-2017-104400 PMC6288698 29070706

[48] Fuscaldo G Genetic ties: are they morally binding? Bioethics 2006;20:64–76. 10.1111/j.1467-8519.2006.00478.x 16770877

[49] Dimond R Social and ethical issues in mitochondrial donation. Br Med Bull 2015;115:173–82. 10.1093/bmb/ldv037 26351372 PMC4562371

[50] Dimond R Techniques of donation: ’three parents', anonymity and disclosure. J Med Law Ethics 2015;3:165–73. 10.7590/221354015X14488767262831

[51] Nuffield Council on Bioethics. Novel techniques for the prevention of mitochondrial DNA disorders: an ethical review. London: Nuffield Council on Bioethics, 2012.

[52] Palacios-González C, Harris J, Testa G Multiplex parenting: IVG and the generations to come. J Med Ethics 2014;40:752–8. 10.1136/medethics-2013-101810 24608087 PMC4215291

[53] Segers S, Mertes H, de Wert G, et al Balancing ethical pros and cons of stem cell derived gametes. Ann Biomed Eng 2017;45:1620–32. 10.1007/s10439-017-1793-9 28091967

[54] Ishii T Potential impact of human mitochondrial replacement on global policy regarding germline gene modification. Reprod Biomed Online 2014;29:150–5. 10.1016/j.rbmo.2014.04.001 24832374

[55] Griffiths D The (Re) production of the genetically related body in law, technology and culture: mitochondria replacement therapy. Health Care Anal 2016;24:196–209. 10.1007/s10728-016-0329-z 27453050 PMC4987392

[56] Sparrow R. A NotSoNew Eugenics. Hastings Cent Rep 2011;41:32–42. 21329104 10.1002/j.1552-146x.2011.tb00098.x

[57] Rulli T What Is the value of three-parent IVF? Hastings Cent Rep 2016;46:38–47. 10.1002/hast.594 27198755

[58] Overall C Why Have Children?: the ethical deabte. Cambridge, Massachusetts: MIT Press, 2012.

[59] Pronatalism PA Geneticism, and art. IJFAB Int J Fem Approaches Bioeth 2017;10:119–47.

[60] Palacios-González C, Allocation R Treatment, disclosure, and mitochondrial replacement techniques: some comments on de melo-martin and harris. Camb Q Healthc Ethics 2017;26:278–87. 28361725 10.1017/S0963180116000876 PMC5964454

[61] Hendriks S, Peeraer K, Bos H, et al The importance of genetic parenthood for infertile men and women. Hum Reprod 2017;32:2076–87. 10.1093/humrep/dex256 28938731

[62] Palacios-González C Mitochondrial replacement techniques: egg donation, genealogy and eugenics. Monash Bioeth Rev 2016;34:37–51. 10.1007/s40592-016-0059-x 27468863 PMC5016536

[63] Newson AJ, Wrigley A Is mitochondrial donation germ-line gene therapy? Classifications and ethical implications. Bioethics 2017;31:55–67. 10.1111/bioe.12312 27973716

[64] Coghlan A ‘3-parent’ baby method already used for infertility. New Sci 2016 https://www.newscientist.com/article/2108549-exclusive-3-parent-baby-method-already-used-for-infertility/ (accessed 5 Dec 2016).

[65] Hamzelou J Exclusive: World’s first baby born with new ‘3 parent’ technique. New Sci 2016 https://www.newscientist.com/article/2107219-exclusive-worlds-first-baby-born-with-new-3-parent-technique/ (accessed 3 Oct 2016).

[66] Hyslop LA, Blakeley P, Craven L, et al Towards clinical application of pronuclear transfer to prevent mitochondrial DNA disease. Nature 2016;534:383–6. 10.1038/nature18303 27281217 PMC5131843

[67] Yamada M, Emmanuele V, Sanchez-Quintero MJ, et al Genetic drift can compromise mitochondrial replacement by nuclear transfer in human oocytes. Cell Stem Cell 2016;18:749–54. 10.1016/j.stem.2016.04.001 27212703 PMC9326498

[68] Boonin D The Non-Identity problem and the ethics of future people. New York, NY: OUP Oxford, 2014.

[69] Dworkin G Is more choice better than less? Midwest Studies in Philosophy 1982;7:47–61. 10.1111/j.1475-4975.1982.tb00083.x

[70] Rose N Powers of freedom: reframing political thought. Cambridge University Press 1999.

[71] Rothman BK The products of conception: the social context of reproductive choices. J Med Ethics 1985;11:188–95. 10.1136/jme.11.4.188 4078857 PMC1375207

[72] Mahowald MB Genes, Women, Equality. 1 edition New York: Oxford University Press, 2000.

[73] Chambers JE Response to ‘Entitlement to cloning’ by Timothy Murphy (CQ Vol 8, No 3) and ‘Cloning and infertility’ by Carson Strong (CQ Vol 7, No 3) May a Woman Clone Herself? Cambridge Quarterly of Healthcare Ethics 2001;10:194–204. 10.1017/S0963180101002110 11302097

[74] Chambers JE Response to ‘Clone alone’ by Carson Strong and ‘Are there limits to the use of reproductive cloning’ by Timothy Murphy (CQ Vol 11, No 1). Cambridge Quarterly of Healthcare Ethics 2002;11:169–79. 10.1017/S0963180102002104 11868422

[75] Murphy TF Response to ‘Cloning and infertility’ by Carson Strong (CQ Vol 7, No 3): entitlement to cloning. Camb Q Healthc Ethics 1999;8:364–8. 10475694

[76] Cloning SC and Infertility. Camb Q Healthc Ethics 1998;7:279–93. 9663349 10.1017/s0963180198703093

[77] Piotrowska M Is ‘assisted reproduction’ reproduction? Philos Q 2017:1–20.

